# Telemedicine comes of age during coronavirus disease 2019 (COVID-19): An international survey of oculoplastic surgeons

**DOI:** 10.1177/1120672120965471

**Published:** 2020-10-17

**Authors:** Elishai Assayag, Maria Tsessler, Lauren M Wasser, Elena Drabkin, Ehud Reich, Yishay Weill, David Zadok, Akshay Gopinathan Nair, Aleza Andron

**Affiliations:** 1 Department of Ophthalmology, Shaare Zedek Medical Center, affiliated with the Hebrew University-Hadassah Medical School, Jerusalem, Israel; 2 Ophthalmic Plastics and Ocular Oncology Services, Aditya Jyot Eye Hospital, Mumbai, Maharashtra, India

**Keywords:** Telemedicine, oculoplastic surgery, COVID-19, survey

## Abstract

**Purpose:**

The Coronavirus disease 2019 (COVID-19) pandemic is an ongoing healthcare crisis that continues its worldwide spread. Ophthalmologists are at high risk of acquiring and transmitting the virus. Telemedicine platforms have evolved and may play an important role in attenuating this risk. For patients, these platforms provide the possibility of clinic consultation without the concerns of a clinic visit. We aimed to assess the utilization of telemedicine by oculoplastics specialists worldwide during the COVID-19 pandemic.

**Methods:**

A 13-item survey was distributed internationally to practicing oculoplastic surgeons. Collected data included demographics, clinical practice variables and perceptions regarding telemedicine. Significance of associations and single survey items was evaluated by Chi-squared and *z*-score of proportions tests, respectively.

**Results:**

The questionnaire was completed by 70 oculoplastic surgeons (54.3% male, mean age 47.3 years, median experience 10 years) from eight countries, practicing in various clinical settings (50.0% hospitals, 45.7% private clinics, 4.3% community clinics). Most respondents reported telemedicine to be an effective tool for oculoplastic consultations (67.1%, *p *= 0.004), while only 12.8% (*p *<* *0.00001) had incorporated this modality into clinical practice prior to the pandemic. Even though a vast majority (98.6%) of participants had limited outpatient activity, most (55.7%) felt unprotected from the virus. Telemedicine had been incorporated by 70.5% (*p* = 0.001) of respondents during the COVID-19 pandemic, whereas most (57.1%) predicted continued use of the modality.

**Conclusion:**

Telemedicine can be effectively and rapidly incorporated into the clinical practice of oculoplastic surgeons during the COVID-19 pandemic. Further research into the most effective utilization of these platforms appears warranted.

## Introduction

The Coronavirus disease 2019 (COVID-19) pandemic continues to be a global health threat.^1^ Severe acute respiratory syndrome coronavirus 2 (SARS-CoV-2)^2^ is highly contagious, exhibits a variable incubation time of 2 to 14 days,^3^ and an estimated global mortality rate of up to 5.7%.^4^

Ophthalmology practice combines high-volume outpatient clinics, surgeries and emergency services. Ophthal-mologists are at prolonged and close contact with patients, many of whom are potential asymptomatic carriers of SARS-CoV-2, placing physicians and their patients at substantial infection risk.^5,6^ Utilization of personal protective equipment (PPE) by ophthalmologists, and limiting clinical activity to emergent care only, were recently emphasized in updates published by the American Academy of Ophthalmology.^7,8^ This new reality is challenging both for physicians who are forced to prioritize patients on a daily basis, and for patients who are postponed until further notice.

Telehealth and telemedicine platforms have previously been utilized and are rapidly growing.^9^ While the technology has existed for some time, full incorporation has been precluded by the large investment required by healthcare systems. The COVID-19 pandemic has resulted in a sudden relaxation of regulations to allow for its expansion,^10^ as evaluating and monitoring patients from distant locations may have great value in minimizing possible exposure to SARS-CoV-2.^11^

The great potential of using telemedicine in ophthalmology has been discussed in recent years.^12^ Clinical evaluations by oculoplastic surgeons appear particularly amenable to the incorporation of telemedicine, due to the highly visual nature intrinsic to the physician-patient encounter.^13^ In this study, we sought to survey oculoplastics specialists worldwide to assess their incorporation of telemedicine into clinical practice during the COVID-19 pandemic.

## Methods

### Data collection

Seventy (70) practicing oculoplastic surgeons from eight countries completed a 13-item online anonymous questionnaire using SurveyMonkey.com. This application is a secure platform for building and managing online surveys (Appendix A). Survey invitations were distributed internationally to oculoplastic surgeons through email and the WhatsApp messenger application. The questionnaire was published online on April 13, 2020 and was available for 7 days. Both objective multiple-choice questions and open questions were utilized. Data collection included demographic information, country of practice, clinical practice attributes, perception of personal safety during the COVID-19 outbreak and opinions regarding telemedicine.

### Statistical analysis

Statistical analysis was performed using GraphPad Prism 6.01 (GraphPad Software, San Diego, CA). Demographic characteristics and properties of clinical settings were presented as mean (standard deviation [SD]) and median (range). Categorical variables were summarized by frequency counts and percentages. In order to analyze single Yes/No questions, a 2-tailed *z*-test for proportions was performed. Associations between two categorical variables were tested in a 2-tailed Chi-squared test. A *P*-value <0.05 was considered statistically significant.

### Ethics and informed consent

This study followed the tenets of the Declaration of Helsinki. The Shaare Zedek Medical Center Institutional Review Board (IRB) approved the study and did not require an individual informed consent from the subjects. The IRB deemed that response to the survey by the ophthalmologists represented an implied consent to participate.

## Results

### Demographic and clinical setups data

The survey was completed by 70 oculoplastic specialists from eight different countries (Table 1).

**Table 1.** Distribution of survey participants by country.

Country	*n* (%)
United States	29 (41.5)
Israel	19 (27.1)
India	14 (20.0)
United Kingdom	3 (4.3)
Russia	2 (2.9)
Canada	1 (1.4)
France	1 (1.4)
Cyprus	1 (1.4)
**Total**	**70 (100)**

Data of survey participants including demographic data, number of patients seen per week and years of experience in oculoplastic surgery are summarized in Table 2. Most respondents stated that their primary clinical setting was a hospital (*n* = 36, 50.0%) or a private clinic (*n* = 32, 45.7%), and only three participants (4.3%) worked in community clinics.

**Table 2.** Demographics, patient volume and years of clinical experience of 70 survey respondents.

Variables	Hospital	Private practice	Community clinic	Total
Age, mean (SD), y	49.7 (13.8)	44.8 (9.6)	46.6 (11.5)	47.3 (11.9)
Gender				
Male, *n* (%)	20 (57.1)	17 (53.1)	1 (33.3)	38 (54.3)
Female, *n* (%)	15 (42.9)	16 (46.9)	2 (66.7)	32 (45.7)
Clinic patients, median (range), *n*/week	65 (40–150)	50 (10–200)	60 (60–80)	60 (10–200)
Experience, median (range), y	13 (1–60)	10 (2–40)	14 (2–27)	10 (1–60)

*n*, number of participants/patients; SD, standard deviation; y, years.

Data is presented by categories of clinical setups.

### Perceptions and previous utilization of telemedicine

The majority of oculoplastic surgeons (67.1%, *z*-score = 2.86, *p* = 0.004) found telemedicine to be an effective tool for oculoplastic consultations (Table 3). No significant difference in this trend was observed between hospital and private practitioners (*χ*^2^ = 2.412, *p* = 0.120).

**Table 3.** Perceptions and previous utilization of telemedicine.

	Hospital	Private practice	Community clinic	Total
Is telemedicine an effective tool for oculoplastic consultations?				
Yes, *n* (%)	26 (74.3)	18 (56.3)	3 (100)	47 (67.1)# **
No, *n* (%)	9 (25.7)	14 (43.7)	0 (0)	23 (32.9)
Were you utilizing telemedicine prior to the COVID-19 outbreak?				
Yes, *n* (%)	6 (17.1)	3 (9.4)	0 (0)	9 (12.9)
No, *n* (%)	29 (82.9)	29 (90.6)	3 (100)	61 (87.1)# ****

*n*, number of participants.

Data is presented in frequency counts and percentages, by categories of clinical setups. Responses were analysed using (#) *z*-test for proportions and Chi-squared test.

** *p *< 0.01, ****p < 0.0001

Nine participants (12.8%) regularly utilized telemedicine in their clinical practice prior to the COVID-19 pandemic (*z*-score = 6.208, *p *<* *0.00001).

### Influences of the COVID-19 outbreak

Data regarding adjustments made in response to the COVID-19 pandemic are depicted in Table 4. A vast majority of respondents (*n* = 69, 98.6%) noted that outpatient clinic activity had been limited to urgent cases only. Most participants remained concerned regarding personal safety, as 39 (55.7%) respondents felt insufficiently protected in terms of PPE and implementation of official COVID-19 guidelines, although this trend did not reach statistical significance (*z*-score = 0.953, *p* = 0.340).

**Table 4.** Clinical setup adjustments due to the COVID-19 pandemic.

	Hospital	Private practice	Community clinic	Total
Was your outpatient clinic activity reduced to urgent cases only?				
Yes, *n* (%)	34 (97.1)	32 (100)	3 (100)	69 (98.6)# ****
No, *n* (%)	1 (2.9)	0 (0)	0 (0)	1 (1.4)
Do you feel sufficiently protected in terms of PPE and guidelines?				
Yes, *n* (%)	18 (51.4)	11 (34.4)	2 (66.7)	31 (44.3)
No, *n* (%)	17 (48.6)	21 (65.6)	1 (33.3)	39 (55.7)
Have you incorporated telemedicine into your practice since the COVID-19 outbreak?				
Yes, *n* (%)	25 (71.4)	24 (75.0)	1 (33.3)	50 (71.4)# **
No, *n* (%)	10 (28.6)	8 (25.0)	2 (67.7)	20 (28.6)
Do you expect telemedicine to be in greater use after the pandemic subsides?				
Yes, *n* (%)	19 (54.3)	19 (59.4)	2 (67.7)	40 (57.1)
No, *n* (%)	16 (45.7)	13 (40.6)	1 (33.3)	30 (42.9)

*n*, number of participants.

Data is presented in frequency counts and percentages, by categories of clinical setups. Responses were analysed using (#) *z*-test for proportions and Chi-squared test.

** *p* < 0.01, ****p < 0.0001.

Since the onset of the COVID-19 pandemic, 50 (71.4%) respondents had incorporated telemedicine into their clinical practice. This majority remained consistent (*n* = 43, 70.5%, *z*-score = 3.186, *p* = 0.001) after exclusion of the respondents who were already using telemedicine prior to the pandemic.

Forty participants (57.1%) predicted greater utilization of telemedicine into their clinical practices after the resolution of the COVID-19. No significant difference was observed between oculoplastic specialists working at hospitals and private clinics (*χ*^2^ = 0.176, *p* = 0.674) in this context.

## Discussion

This international survey demonstrated that during the COVID-19 outbreak, a majority of oculoplastic surgeon respondents initiated use of telemedicine, whereas prior to the outbreak only 12.8% had utilized this method.

One can hypothesize many reasons for this abrupt change. One reason is that the pandemic provided the impetus to break out of established patterns of practice. During the COVID-19 pandemic, oculoplastic surgeons were faced with a new reality: the ophthalmologic exam, which requires close contact between the patient and physician, became a potential threat to their patients and to themselves^14^. The presence of virus in the tears of 2 of 30 patients as described in one report^15^ as well as the documentation of transmission through asymptomatic carriers^16^ revealed the possibility of coronavirus infection through routine ophthalmologic practice. In light of these revelations during the outbreak, it is not surprising that many oculoplastic surgeons stated that they did not feel sufficiently protected during the physical examination of patients and reduced their activities to the examination of urgent patients exclusively. A second reason was that a large percentage of patients themselves were missing even semi-urgent clinic appointments forcing physicians to attempt to accommodate their needs.^17^

A third reason for the abrupt sudden incorporation of telemedicine was the previous lack of infrastructure. As a result of the pandemic, the urgent need for remote visits overcame the bureaucratic challenge and the system suddenly became amenable to the utilization of these modalities. Legislation and coding began to support the platform in many countries, specifically in the United States where telemedicine was not previously reimbursed. This changed abruptly at the outset of the pandemic.^18^ The disadvantages of telemedicine are clearly evident and may account for a percentage of respondents not expecting to incorporate the modality as part of their practice after the pandemic. Video conferencing technology may not be available in many physician offices. Additionally, even with the use of videoconferencing, aspects of the physical examination become more cumbersome and at times inflections in speech and mannerisms may be more easily seen in person. Nonetheless, the majority of respondents in the field of oculoplastics judges the modality favourably and did expect to see telemedicine incorporated into their practices.

The limitations of this survey include its small sample size and that it spanned a few countries with highly diverse oculoplastic surgical practice patterns, health and legal systems. An exploratory survey such as this cannot be expected to describe in detail nor adequately predict the potential of oculoplastic surgical telemedicine practice potential in any of the countries represented. This survey, however, can indicate that the modality of telehealth and telemedicine is worthy of serious evaluation and consideration as a tool for worldwide oculoplastic surgeons and their healthcare systems.

In conclusion, this study demonstrated effective utilization of telemedicine by oculoplastic specialists during COVID-19, a modality that had not been previously utilized by many of them. An international survey of oculoplastic surgeons during the COVID-19 pandemic showed that respondents were able to incorporate telemedicine into their practices during the pandemic. The respondents also expected that telemedicine would be a useful modality in clinical practice after the pandemic subsides. As such, regulators and platform developers are urged to consider wider distribution of telemedicine modalities for the oculoplastic surgical subspecialty and to encourage further exploration of their potential.

## Supplemental Material

Appendix_A – Supplemental material for Telemedicine comes of age during coronavirus disease 2019 (COVID-19): An international survey of oculoplastic surgeons Click here for additional data file. Supplemental material, Appendix_A for Telemedicine comes of age during coronavirus disease 2019 (COVID-19): An international survey of oculoplastic surgeons by Elishai Assayag, Maria Tsessler, Lauren M Wasser, Elena Drabkin, Ehud Reich, Yishay Weill, David Zadok, Akshay Gopinathan Nair and Aleza Andron in European Journal of Ophthalmology
