# Supplementary material for: Telemedicine comes of age during coronavirus disease 2019 (COVID-19): An international survey of oculoplastic surgeons
Source: Eur J Ophthalmol. 2020 Oct 17;31(6):2881–5. doi: 10.1177/1120672120965471 (PMC8606803; doi:10.1177/1120672120965471)
Supplement: Appendix_A – Supplemental material for Telemedicine comes of age during coronavirus disease 2019 (COVID-19): An international survey of oculoplastic surgeons [file Appendix_A.pdf]

Appendix A.-Telemedicine Utilization by Oculoplastic Surgeons during the COVID-19 Pandemic

1. What is your age?

____

2. What is your gender?

- o Female
- o Male

3. How many years have you been practicing as an Oculoplastic surgeon?

_____

4. What country do you practice in?

_____

5. What is your clinical setting? If you practice in more than one setting, please refer to the primary setting:

- o Hospital
- o Community clinic
- o Private practice

6. Approximately how many patients do you usually see in an outpatient setting per week?

_____

7. In your opinion, is telemedicine an effective tool for oculoplastic consultations?

- Yes
- No

8. Were you utilizing telemedicine in your practice prior to the COVID-19 outbreak?

- Yes
- No

9. Given the current status of COVID-19 in your country and setting, do you feel sufficiently protected in terms of personal protective equipment and implementation of COVID-19 guidelines?

- Yes
- No

10. Since the COVID-19 outbreak, was your outpatient clinic activity reduced or limited to urgent cases only?

- Yes
- No

11. Since the COVID-19 outbreak, have you incorporated telemedicine into your clinical practice?

- Yes
- No

12. Do you expect telemedicine to be in greater use in your practice after the COVID-19 pandemic subsides?

- o Yes
- o No

13. Please feel free to leave any other comments or suggestions:
